# The Influence of Verbal Suggestion on Post-Needling Soreness and Pain Processing after Dry Needling Treatment: An Experimental Study

**DOI:** 10.3390/ijerph18084206

**Published:** 2021-04-15

**Authors:** Eleuterio A. Sánchez Romero, Tifanny Lim, Jorge Hugo Villafañe, Gurvan Boutin, Victor Riquelme Aguado, Aitor Martin Pintado-Zugasti, José Luis Alonso Pérez, Josué Fernández Carnero

**Affiliations:** 1 Musculoskeletal Pain and Motor Control Research Group, Faculty of Health Sciences, Universidad Europea de Madrid, 28670 Madrid, Spain; tifanny94@live.fr (T.L.); boutin.gurvan@gmail.com (G.B.); 2 Department of Physiotherapy, Faculty of Biomedical and Health Sciences, Universidad Europea de Madrid, 28670 Madrid, Spain; 3 IRCCS Fondazione Don Carlo Gnocchi, 20141 Milan, Italy; 4 Department of Basic Health Sciences, Faculty of Health Sciences, Rey Juan Carlos University, 28922 Madrid, Spain; victor.riquelme@urjc.es; 5 Department of Physical Therapy, Faculty of Medicine, CEU-San Pablo University, 28040 Madrid, Spain; martinpintado@ceu.es; 6 Musculoskeletal Pain and Motor Control Research Group, Faculty of Health Sciences, Universidad Europea de Canarias, La Orotava, Tenerife, 38300 Canary Islands, Spain; 7 Rey Juan Carlos University, Department of Physical Therapy, Occupational Therapy, Rehabilitation and Physical Medicine, 28032 Madrid, Spain; 8 La Paz Hospital Institute for Health Research, IdiPAZ, 28046 Madrid, Spain; 9 Grupo Multidisciplinar de Investigación y Tratamiento del Dolor, Grupo de Excelencia Investigadora URJC-Banco de Santander, Alcorcón, 28933 Madrid, Spain

**Keywords:** dry needling, pain, trigger points, psychological factors

## Abstract

Background: It remains unclear as to whether verbal suggestions and expectancies can influence the perception of post-needling soreness. The aim of this study was to analyze the effects of verbal suggestions on post-needling soreness after dry needling of the trapezius muscle. Methods: This study is a randomized controlled trial including healthy subjects randomly assigned to one of three groups receiving different verbal suggestions about the effects of dry needling and the occurrence of post needling soreness (positive, negative, or neutral). Then, dry needling on a latent trigger point of the upper trapezius muscle was performed and the following outcomes were measured immediately after, 24, 48, and 72 h, and one week after the intervention: post-needling soreness intensity, pressure pain threshold (PPT), temporal summation (TS) and conditioned pain modulation (CPM). Results: Seventy-three consecutive participants were screened and 42 participants (12 men and 30 women, aged: 24 ± 8 years old) were eligible and finished the study protocol. The results showed that verbal suggestion did not influence the perception of post-needling soreness, since there were no differences between groups (*p* < 0.05) on the intensity of post-needling soreness or tenderness over a one-week follow-up. Moreover, verbal suggestion did not associate with changes in sensorimotor variables of TS and CPM. Conclusions: The induction of different types of expectations through verbal suggestion does not influence the perception of acute pain perceived during the performance of a deep dry needling technique and post-needling pain or soreness after deep dry needling on a latent upper trapezius myofascial trigger point (MTrP).

## 1. Introduction

Dry needling is frequently directed at myofascial trigger points (MTrPs), which are hypersensitive nodules in taut bands present in skeletal muscles associated with multiple pain conditions such as shoulder pain [1], mechanical neck pain [2], tension-type headaches [3], temporomandibular disorders [4] or knee pain [5]. Recent reviews and meta-analyses on the effectiveness of MTrPs dry needling have suggested or recommended dry needling for the treatment of lateral epicondylalgia [6], patellofemoral pain [7], and neck pain [8].

Significant adverse events of dry needling are rare, but mild adverse effects, including pain during or after the treatment, are very common [9]. Pain after dry needling treatment is known as post-needling soreness and it is associated with intramuscular injury, edema, and inflammation produced by the repeated insertions of the needle [10,11,12].

Most patients report some degree of post-needling soreness after MTrP dry needling, and so this phenomenon has been the focus of multiple investigations to describe post-needling soreness characteristics or evaluate the effectiveness of additional therapies for minimizing its perception [12,13]. The real clinical relevance of post-needling soreness remains unclear and it has been suggested that patient expectations and the information provided to the patient about post-needling soreness may play a role. Therefore, further research has been recommended to investigate the importance of the information provided to the patient or the patients’ beliefs about post-needling soreness [14,15].

Previous research on acute procedural pain (pain during or directly after a medical procedure, e.g., postoperative pain) observed that verbal suggestion interventions to induce analgesic expectations relieved patients’ procedural pain, suggesting they could be used to optimize the effectiveness of standard analgesic treatments in clinical practice [16]. Opposite, inducing negative expectations regarding adverse effects, such as verbal suggestions of potential side effects may, in itself, lead to the experience of aversive side effects [17].

A recent study [15] investigated the impact of physical therapists’ verbal suggestion about post-needling soreness (positive, negative, or no suggestion) on the outcomes of dry needling regarding neck pain, widespread pressure pain sensitivity, and neck functional disability. All groups showed similar improvements regardless of the type of verbal suggestion received, indicating that the impact of positive or negative suggestions was negligible for the effectiveness of MTrP dry needling on the treatment of neck pain. However, it remains unclear whether verbal suggestion and expectancies can influence the perception of post-needling soreness. We hypothesize that negative verbal suggestions about post-needling soreness may associate with higher post-needling soreness intensity perception and short-term changes in temporal summation (TS) and conditioned pain modulation (CPM) and pressure pain threshold (PPT) due to nocebo effects.

Therefore, the aim of this study was to examine the effect of verbal suggestion (positive, neutral, or negative) on post-needling soreness in healthy subjects after dry needling over trapezius muscle.

## 2. Materials and Methods

### 2.1. Study Design

**Figure 1:**
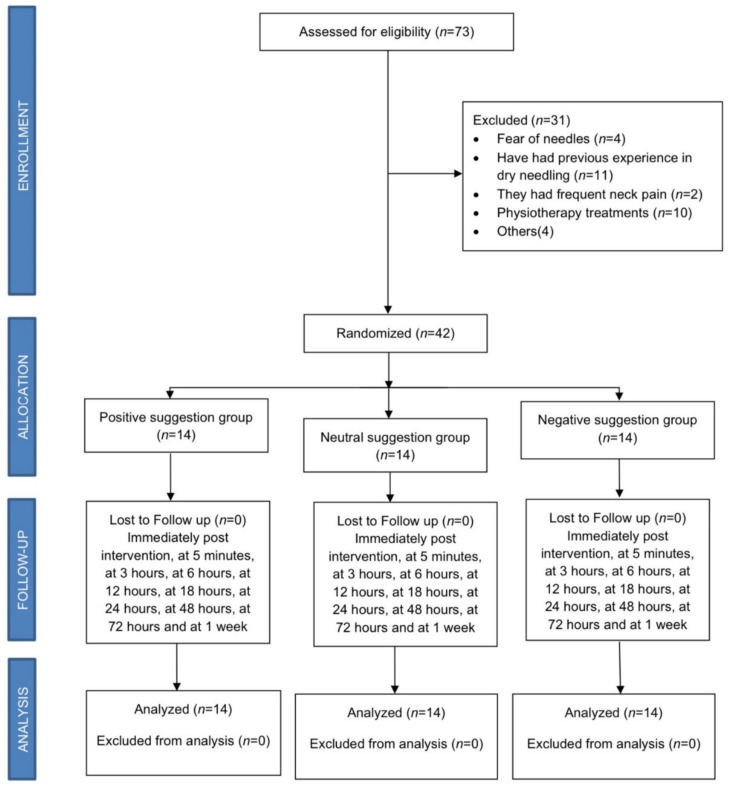
CONSORT 2010 flow diagram.

We conducted a multicenter, single-blind, randomized, controlled clinical trial (RCT). Informed consent was obtained from all patients, and procedures were conducted according to the Declaration of Helsinki and approved by a Local Ethical Committee of the Rey Juan Carlos University, Madrid, Spain (0504201708117), and registered in ClinicalTrials.gov (NCT04571827). All subjects signed an informed consent prior to their inclusion, and the study was developed according to CONSORT 2010 Statement: updated guidelines for reporting parallel group randomised trials (CONSORT 2010 flow diagram, Figure 1).

### 2.2. Subjects

From October and December 2020, forty-two healthy participants residing in the Community of Madrid were recruited to participate in this study at Rey Juan Carlos University and the European University of Madrid.

Inclusion criteria for this study were: (1) age between 18 and 62 years; (2) presented at least one latent MTrP in the upper trapezius muscle; (3) speaking and understanding Spanish correctly; (4) no previous experience in dry needling treatment. We excluded participants with: neurological signs or symptoms; a history of injury, fracture, or previous spinal surgery; a history of musculoskeletal and/or rheumatological diseases; insurmountable fear of needles; coagulation disorders; corticosteroids infiltration, or use of local anesthetics for one year before the study; taking analgesic or anti-inflammatory medication the week before the study.

### 2.3. Procedures

Participants were randomly assigned to three groups: positive, negative, and neutral verbal suggestion. Each group received a different verbal suggestion to attempt to influence participant expectation. The positive expectation group was told the sentence: “it is a very effective technique that achieves excellent results in the improvement of the cervical muscles”. The negative expectation group was told the sentence: “this is a technique that will cause discomfort in the area of intervention of the cervical muscles after applying it”. The neutral expectation group was told the sentence: “it’s a physical therapy technique used to treat neck pain and we’re investigating its effects”.

Randomization was conducted using GraphPad Software’s QuickCals application (La Jolla, CA, USA). All participants were examined to diagnose latent MTrP in the upper trapezius muscle by palpation using a pincer grasp between the thumb and the index fingers. The presence of latent MTrP was defined on the basis of finding, by palpation, a taut band, a palpable nodule in the taut band, and a hypersensitive point [18].

Then, the dry needling procedure was performed based on the method described by Hong [19]. After cleaning the area with an antiseptic solution, the therapist held firmly the MTrP using the same pincer grasp and perforated the muscle fiber with a solid filament needle (0.26 × 40 mm^2^). This consisted of 15 instances [20] of manipulating the needle upwards and downwards inside the muscle. After removing the needle, a soft compression with a cotton swab was applied to reduce the possible appearance of bleeding. The number of needle insertions, the number of twitch responses, and the presence or absence of bleeding were recorded.

### 2.4. Description of Outcome Variables

#### 2.4.1. Post-Needling Soreness Visual Analog Scale (VAS)

Post-needling soreness was assessed using the visual analog scale (VAS) for pain. This tool is a 100 mm line that measures pain intensity. The left end of the line represents the absence of pain, while the right end represents the worst pain imaginable. The numerical pain intensity scale adds numerical graduation where 1 is no pain and 10 is the worst pain imaginable. The confidence and reliability of this scale have been approved and validated in different studies [21].

#### 2.4.2. Pressure Pain Threshold (PPT)

A handheld pressure algometer (Model FDIX, Wagner InstrumentMark, USA) with a 1 cm diameter flat rubber probe was used to evaluate the PPT before and during the conditioning stimulus. With a pressure algometer, we measured pressure pain detection on the upper muscle fibers of the trapezius of each participant [22]. To improve accuracy, the researcher in charge of taking this measurement precisely marked the area of pain with a marker. Each measurement was performed three times.

#### 2.4.3. Temporal Summation (TS)

TTS was evaluated 5 min before CPM performance using a handheld pressure algometer (Model FDIX, Wagner InstrumentMark), with a 1 cm diameter flat rubber probe. TS was elicited with 10 pressure stimulation at pressure pain detection threshold intensity [23].

#### 2.4.4. Conditioned Pain Modulation (CPM)

The CPM value is the result of the subtraction of the value of the PPT without stimulus from the value of the PPT during the conditioning stimulus [24].

Measures were taken pre-intervention, immediately post-intervention, and at 5 min, 3 h, 6 h, 12 h, 18 h, 24 h, 48 h, 72 h, and 1 week, post-intervention.

Participants had to complete the questionnaire of psychological and disability variables on post-needling pain and expectations before intervention: Neck Disability Index (NDI) [25], State-Trait Anxiety Inventory (STAI) [26], Beck Depression Inventory (BDI-II) [27,28], Pain Catastrophizing Scale (PCS) [29,30,31,32], Tampa Scale for Kinesophobia (TSK) [33], Pain Anxiety Symptoms Scale (PASS-20) [34,35,36], and Fear of Pain Questionnaire (FPQ-III) [37] were assessed.

### 2.5. Sample Size

The sample size was estimated with the program G*Power 3.1.7 for Windows (G*Power from University of Dusseldorf, Germany) [38]. The post-needling soreness was chosen as the principal variable. We considered three groups and two measurements for primary outcomes to obtain 95% statistical power (1- β error probability) with an α error level probability of 0.05 using analysis of variance (ANOVA) of repeated measures, within-between interaction, and an effect size moderate (effect size *f* = 0.25). This generated a sample size of a total of 35 participants plus an estimated 20% loss in follow-up, yielding a total of 42 participants (14 per group).

### 2.6. Data Analysis

We performed the data analysis with the Statistics Package for Social Science (SPSS 25.00, IBM Chicago, IL, USA), employing a 95% confidence interval and considering all values with a *p*-value inferior to 0.05 to be statistically significant. The descriptive statistics for continuous variables are presented as mean ± standard deviation and the 95% confidence interval. We performed a repeated-measures ANOVA to study the effect of the between-subject factor ‘intervention group’ with 3 categories; Positive suggestion group (PSG), Neutral suggestion group (NSG), Negative suggestion group (NeSG) and the within-subject factor “time”, also with two categories (pre and post) on the dependent variables. We calculated the partial eta squared as a measure of effect size (strength of association) for each main effect and interaction in the ANOVAs, with 0.01–0.059 representing a small effect, 0.06–0.139 a medium effect, and >0.14 a large effect. We performed a post hoc analysis with Bonferroni correction in the case of significant ANOVA findings for multiple comparisons between variables. The effect size is calculated using the partial eta squared (ηp^2^) when significant. An effect size greater than 0.8 was considered large, around 0.5, moderate, and less than 0.2, small.

## 3. Results

### 3.1. Demographic and Clinical Data of Subjects

Seventy-three consecutive participants were screened and 42 participants (12 men and 30 women, aged: 24 ± 8 years old) were eligible and agreed to participate. Figure 1 shows the recruitment and retention of participants through the trial. Baseline features of groups were similar for all variables Table 1.

### 3.2. VAS Post-Needling Soreness

**Figure 2:**
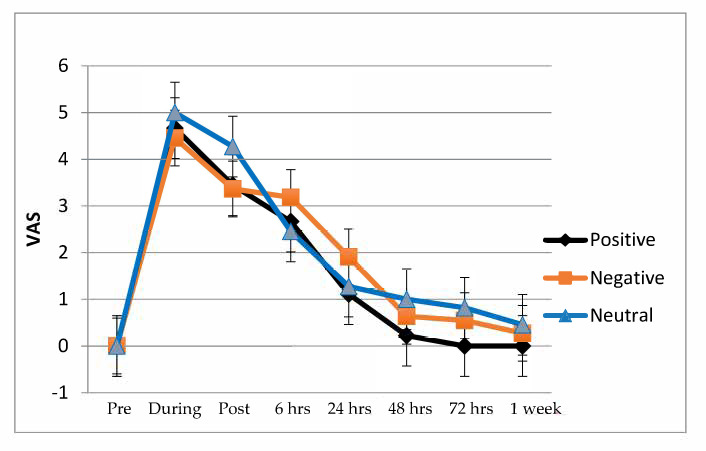
Post-needling soreness VAS (Visual Analog Scale) evolution in each group of positive, negative or neutral suggestion between pre-treatment data and 24 h, 48 h, 72 h and 1 week after treatment.

VAS data for post-needling soreness are presented Figure 2 and Table 2. The ANOVA did not reveal significant group x time interaction (*F* = 0.44; *p* = 0.943). All groups improved over time (*F* = 53.71; *p* = 0.0001; ηp^2^ = 0.592). Also, a large within-group effect sides (*d* > 1) was found between pre-treatment data and 24 h, 48 h, 72 h and 1 week. Between-groups, effect sizes were less at all follow-up periods (*d* < 0.2).

### 3.3. Pressure Pain Threshold

**Figure 3:**
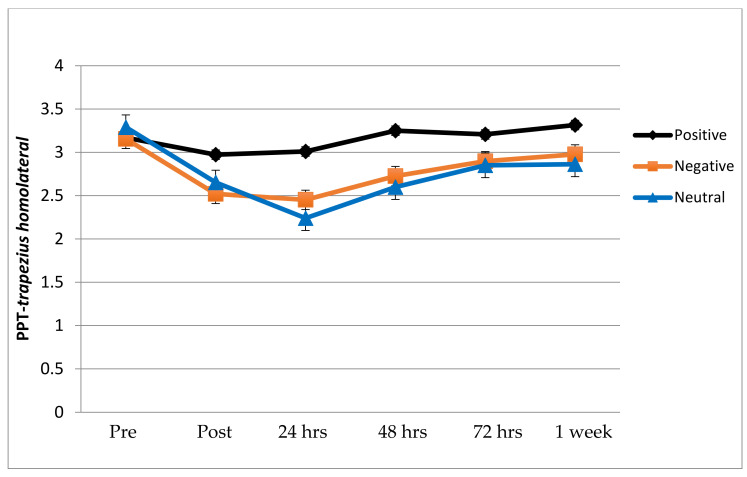
Pressure Pain Threshold (PPT) evolution in each group (homolateral trapezius muscle).

PPT data for the two sites are presented in Figure 3 and Table 3. The ANOVA revealed no statistically significant group x time (all, *p* > 0.05) interactions. The ANOVA did reveal significant effects for the time factor (*F* = 5.776; *p* = 0.0001; ηp^2^ = 0.138 and *F* = 5.559; *p* = 0.0001; ηp^2^ = 0.217) for PPT over the homolateral and contralateral trapezius respectively. The within-group effect sizes were between moderate and less at all follow-up periods (*d* < 0.9).

### 3.4. Temporal Summation (TS) and Conditioned Pain Modulation (CPM)

For TS and CPM, the ANOVA revealed no significant group x time (*F* = 0.682; *p* = 0.609 and *F* = 0.481; *p* = 0.757) or time factor (*F* = 0.688; *p* = 0.699 and *F* = 1.084; *p* = 0.379) interactions, respectively. TS and CPM data are presented in Table 3.

## 4. Discussion

Our results showed that verbal suggestion did not influence the perception of post-needling soreness, since there were no differences between groups on the intensity of post-needling soreness or tenderness over a one-week follow-up. Moreover, verbal suggestion did not associate with changes in sensorimotor variables of TS and CPM.

### 4.1. Induced Expectations and Post-Needling Soreness

To the authors’ knowledge, no previous research has specifically investigated whether verbal suggestion or induced expectations influence the experience of post-needling soreness. A recent study [15] has observed that verbal suggestion about post-needling soreness (positive, negative, or no suggestion) did not influence the outcomes of dry needling regarding neck pain, disability, and widespread pressure pain sensitivity, indicating that the impact of verbal suggestion was negligible for the effectiveness of MTrP dry needling for the treatment of neck pain.

In the systematic review by Boerner et al. [39] regarding psychological interventions for reducing pain from common needle procedures, they observed evidence that signaling the patient about the impending pain that may be experienced during procedures such as venipuncture was associated with increased levels of pain when compared with only signaling to the patient about the procedure (without reference to pain or discomfort). Although these results are not directly comparable with those of the present work, our research did not observe an influence of informing the patient about the occurrence of post-needling soreness (negative expectation group) when compared with suggesting the positive effects of the intervention (positive expectation group) or not mentioning any soreness or positive effects associated to the intervention (neutral expectation group). It can be hypothesized that the influence of psychological factors during venipuncture processes plays a more important role in the perception of procedural pain compared with dry needling and the intensity of post-needling soreness. Previous research has not observed a relationship between increased levels of psychological factors and an augmented perception of post-needling soreness [40]. In addition, participants in our study had never received dry needling before, so they may not have any expectations of post-needling soreness, occurrence, or intensity before the negative verbal suggestion was introduced. This may be different in other common needle procedures such as venous cannulation or venipuncture.

Regarding the influence of verbal suggestion on the outcomes of dry needling, our study did not show any effects on TS and CPM. To our knowledge, no previous research had investigated the influence of verbal suggestion before dry needling on TS and CPM. However, various studies have analyzed whether verbal suggestions or expectations influence the outcomes of other interventions such as manual therapy.

Previous research by Bialosky et al. [41] on the influence of expectations on the outcomes of manual therapy observed that negative expectations caused a nocebo effect, while the positive and neutral ones caused the expected clinical results. These results were not observed in our study, and this could be due to the fact that the technique performed in the study by Bialosky et al. is a technique in which the patient does not know whether or not he/she is going to feel pain and, therefore, it is easier to influence through expectation causing a nocebo effect, while the dry needling technique, to a greater or lesser extent, the patient can expect that the procedure will associate with pain, regardless of the verbal suggestion of the clinician.

Although we cannot directly compare, our results are not in line with those found when applying manual therapy in patients with neck pain [42], in which they observed that patients who received positive or neutral verbal suggestions improved pain intensity and mechanical hyperalgesia, even though the positive effect lasted only one week. These differences with our study can be explained because they did not perform invasive treatment and because they were performed with subjects in pain. However, in our study, we did not observe that the group who received negative suggestions had worsened levels of CPM or TS or post needling soreness, like the same outcomes of this study [42].

Although we did not find differences between the different verbal suggestions, a meta-analysis found a moderate effect size for pain relief in favor of verbal suggestions [16]. However, the effect size is large when the pain is produced by acute painful procedures and not so large in chronic pain. Those findings, together with the fact that in previous studies have found effects of verbal suggestions, varied depending on the route of administration of the therapy, with larger effects in cases involving invasive treatments versus non-invasive treatments [43,44], which could partly explain why, in our study, no differences were observed between the different types of suggestions, since neither tapping, being a painful technique and on the other hand being an invasive therapy, could activate endogenous modulation systems. In contrast, other studies in patients with osteoarthritis who received verbal suggestions associated with acupuncture treatment, but applied to acupuncture points other than those necessary to produce an effect, produced a greater improvement in patients than the group who received a neutral suggestion [45] which was maintained for 3 months. Even a recent narrative review found that positive verbal suggestions have favorable effects on osteoarthritis pain and low back pain [46]. In relation to conditioned modulation, a recent study has found that inducing positive affect reverses nocebo hyperalgesia by applying the conditioned stimulus [47].

The plausible mechanisms that explain the effects of the verbal suggestions are on the one hand anxiety reductions [48,49], but a recent meta-analysis did not demonstrate the effects on anxiety. Another study conducted by Vase et al. [50] found that anxiety reduction was observed after suggestions [50]. In this sense, we cannot support or refute these theories, since, in our study, we only measured anxiety at baseline, and our subjects did not show much anxiety, without exceeding the cut-off line of 39−40 points, with them not scoring more than 27.7 points. Other neurophysiological effects such as reduction of heart rate [51] and c-reactive protein [52], but not cortisol [53], were observed after verbal suggestions [51,52,53,54,55]. In contrast, in a study using manual therapy, an increase in cortisol was observed in the groups that received neutral and negative verbal suggestions, but these changes in cortisol were not associated with improvements or worsening of the patients’ pain [42]. On the other hand, it has been proven that when we introduce positive verbal suggestions, they activate the same brain areas related to pain relief as a pharmacological treatment for pain [56]. It has been proven that when we introduce positive verbal suggestions they activate the same brain areas related to pain relief as a pharmacological pain treatment. We also know that verbal suggestions have been shown to activate opioid and dopaminergic and endocannabinoid pathways [57]. But despite all these effects demonstrated, in our study we have not shown that verbal suggestions can activate a placebo or nocebo pathway. Therefore, incorporating words that induce positive expectations does not influence the mechanical hyperalgesia and post-needling pain of the tested subjects. On the other hand, it has been shown that adding pain education to patients receiving dry needling improves the positive effects on pain and psychological variables such as kinesiophobia [58].

One aspect that was not assessed in this study, and that is important to mention for future research, is the patient’s beliefs about post-needling pain and the dry needling technique. In another manual therapy study by Bishop et al. [59], the patient’s own expectations about the technique that they are going to receive were evaluated, so that when the patient received the intervention they want to receive, the subjects report greater satisfaction with the treatment, which could increase adherence to it, although there were no significant differences in the improvement of hypoalgesia compared to receive a different manual therapy technique that produces similar effects.

On the other hand, it has been found that patients who have the greatest desire to relieve their pain are possibly the most influenced by verbal suggestions, and in this sense, we have not controlled for this variable [60].

The results of this study question whether the information provided to the patient about post-needling soreness influences its perception. However, new research with larger pain populations is needed to further investigate these influences. Our results also may be relevant when discussing what is the real clinical relevance that post-needling soreness has for patients since it remains unclear.

We think that our results are not transferable to the pain population, because they are healthy subjects with no previous pain and the effects are different from those of patients with pain because they usually have lower expectations due to the failure of previous treatments [61].

### 4.2. Limitations

The present study has several limitations. First, we did not include placebo control groups using sham dry needling to compare them with the experimental groups. Second, although the verbal suggestion was used, we did not directly assess the patient’s own beliefs or expectations about post-needling soreness or about the deep dry needling technique to see how the patient’s expectations were induced by the therapist’s verbal suggestion. Third, post-needling soreness was assessed after dry needling of latent MTrPs. Further research should investigate the effects of verbal suggestion and expectancies when performing dry needling in active MTrPs present in the pain population. Fourth, the results of the study are limited to the specific verbal suggestion statements and vocal tone used. The characteristics of the statements and words used may associate with different changes in expectations and post-needling soreness perception. Fifth, the sample size limits the generalization of results. Further research should investigate the influence of verbal suggestions and expectancies on post-needling pain in larger populations.

More research needs to be done in the future, taking into account the elements of verbal suggestion, mediating factors such as patients’ expectations, physiological and psychological responses such as anxiety or attention and moderating factors such as previous experience, desire for pain relief, and previous history of pain treatment received, because all of these factors can provide information on why and how verbal suggestions can have an impact. It is also necessary to do this research on pain patients and not on healthy subjects to see what influences they can have when a person suffers from chronic pain.

Finally, we believe it would be useful to recommend the inclusion of manipulation controls in future studies to learn participants’ perceptions of the different verbal instructions given.

## 5. Conclusions

The induction of different types of expectations through verbal suggestion does not influence the perception of acute pain perceived during the performance of a deep dry needling technique and post-needling pain soreness after deep dry needling on a latent upper trapezius MTrP.

Dry needling and verbal suggestion did not show influences on somatosensory variables of mechanical hyperalgesia (PPT), the descending inhibitory pain system (CPM), and neuronal hyperexcitability (TS), and it seems that negative verbal suggestion does not have a nocebo effect.

## Figures and Tables

**Table 1:** Characteristics of patients at baseline.

Characteristics	Group			
	Positive Suggestion	No Suggestion	Negative Suggestion	*p*-Value
	(*n* = 14)	(*n* = 14)	(*n* = 14)	
Age (yr), mean (SD)	23 (6)	23 (5)	27 (12)	>0.05
Gender (*n*), female (%)	13 (58)	9 (6)	10 (52)	>0.05
TS, mean (SD)	2.4 (1.5)	2.4 (1.5)	2.5 (1.4)	>0.05
CPM, mean (SD)	4.9 (2.7)	4.1 (1.5)	4.5 (0.6)	>0.05
NDI, mean (SD)	5.3 (2.6)	5.3 (3.5)	5.3 (6.9)	>0.05
STAI, mean (SD)	25.0 (8.4)	27.7 (8.8)	27.0 (4.1)	>0.05
BDI-II, mean (SD)	6.3 (1.7)	5.3 (1.3)	4.9 (1.5)	>0.05
PCS, mean (SD)	7.9 (3.8)	8.5 (10.3)	8.6 (10.1)	>0.05
TSK, mean (SD)	10.3 (5.0)	10.1 (5.5)	10.2 (7.6)	>0.05

Temporal Summation (TS); Conditioned pain modulation (CPM); Neck Disability Index (NDI); State-Trait Anxiety Inventory (STAI); Beck Depression Inventory (BDI-II); Pain Catastrophizing Scale (PCS); Tampa Scale for Kinesophobia (TSK) and Pain Anxiety Symptoms Scale (PASS-20).

**Table 2:** ANOVA comparing effects between positive suggestions vs. negative suggestions vs. neutral suggestions on post-needling soreness for VAS.

Vas	Positive Suggestions	Negative Suggestions	Neutral Suggestions	Time Effect Sizes	Group
VAS during	4.8 ± 2.9	4.5 ± 2.3	4.8 ± 3.0	-	1.00
VAS post	3.8 ± 1.8	3.1 ± 2.2	4.2 ± 2.5	0.2 to 0.6	1.00
VAS at 6 h	3.8 ± 1.8	2.7 ± 2.1	2.6 ± 2.3	0.4 to 0.8	1.00
VAS at 24 h	1.7 ± 1.8 *	1.4 ± 1.1 *	1.2 ± 2.2 *	1.3 to 1.7	1.00
VAS at 48 h	0.7 ± 1.2 *	0.5 ± 0.9 *	0.9 ± 2.2 *	1.5 to 2.3	1.00
VAS at 72 h	0.0 ± 0.0 *	0.4 ± 0.9 *	0.7 ± 2.0 *	1.6 to 2.3	1.00
VAS at 1 week	0.4 ± 1.4 *	0.3 ± 0.8 *	0.4 ± 1.4 *	1.9 to 2.4	1.00

VAS: Visual Analog Scale. * Indicates statistical significance *p* < 0.05.

**Table 3:** ANOVA comparing effects between positive suggestions vs. negative suggestions vs. neutral suggestions on post-needling soreness for PPT, CPM, and TS.

	Positive Suggestions	Negative Suggestions	Neutral Suggestions	Time Effect Sizes	Group
PPT_homolateral side					
PPT-Post	2.8 ± 1.1	2.3 ± 0.8	2.5 ± 0.9	-	0.4
PPT-24 h	2.6 ± 0.8	2.5 ± 1.2	2.3 ± 1.2	0.1 to 0.2	1.0
PPT-48 h	2.8 ± 0.9	2.9 ± 0.9	2.8 ± 1.1	0.1 to 0.6	1.0
PPT-72 h	3.1 ± 0.9	2.8 ± 0.9	3.0 ± 1.3	0.3 to 0.8	1.0
PPT-1 week	3.2 ± 0.8	3.1 ± 0.7	3.3 ± 1.5	0.4 to 0.9	1.0
PPT-contralateral side					
PPT-Post	2.1 ± 1.1	2.7 ± 0.9	2.9 ± 1.5	-	0.5
PPT-24 h	2.6 ± 1.1	2.9 ± 0.8	3.6 ± 1.7	0.2 to 0.6	0.3
PPT-48 h	2.9 ± 1.1	2.7 ± 0.5	3.7 ± 1.5	0.1 to 0.7	0.2
PPT-72 h	3.0 ± 0.8	2.8 ± 0.4	3.7 ± 1.4	0.1 to 0.9	0.1
PPT-1 week	3.3 ± 1.1	3.1 ± 0.9	3.9 ± 1.7	0.4 to 0.6	0.5
Conditioned pain modulation					
CPM-24 h	1.0 ± 0.8	0.3 ± 0.6	0.6 ± 0.5	-	0.6
CPM-48 h	0.8 ± 0.5	0.1 ± 0.1	0.8 ± 0.8	0.1 to 0.3	0.5
CPM-72 h	1.5 ± 1.0	0.3 ± 1.5	0.6 ± 0.9	0.1 to 0.6	0.5
CPM-1 week	1.0 ± 0.0	0.0 ± 0.0	1.0 ± 0.7	0.1 to 0.1	0.6
Temporal summation					
TS-24 h	0.3 ± 0.5	0.7 ± 0.6	−0.2 ± 1.1	-	1.00
TS-48 h	0.5 ± 2.1	0.3 ± 1.2	0.0 ± 1.0	0.1 to 0.3	1.00
TS-72 h	0.8 ± 0.5	0.3 ± 0.6	0.2 ± 0.8	0.1 to 0.4	1.00
TS-1 week	1.0 ± 0.8	0.3 ± 0.6	−0.2 ± 0.8	0.1 to 0.4	0.87

Temporal Summation (TS); Conditioned pain modulation (CPM); Pressure Pain threshold.

## Data Availability

The data presented in this study are available on request from the corresponding authors.
